# Circulating Levels of Inflammatory Markers in Intrauterine Growth Restriction

**DOI:** 10.1155/2010/790605

**Published:** 2010-06-03

**Authors:** Theodora Boutsikou, George Mastorakos, Marialena Kyriakakou, Alexandra Margeli, Demetrios Hassiakos, Ioannis Papassotiriou, Christina Kanaka-Gantenbein, Ariadne Malamitsi-Puchner

**Affiliations:** ^1^Neonatal Division, Second Department of Obstetrics and Gynecology, University of Athens, 11528 Athens, Greece; ^2^Department of Clinical Biochemistry, “Aghia Sophia” Children's Hospital, 11527 Athens, Greece; ^3^First Department of Pediatrics, University of Athens, 11527 Athens, Greece

## Abstract

We aimed to investigate possible alterations in circulating levels of the perinatal stress markers high sensitivity (hs)-CRP, PAI-1, and S100B—probably reflecting brain and adipose tissue inflammation—in intrauterine growth-restricted-(IUGR) and appropriate-for-gestational-age-(AGA) pregnancies, given that these groups differ in fat mass and metabolic mechanisms involving aseptic inflammation. Serum hs-CRP, PAI-1, and S100B levels were measured in 40 mothers, and their 20 AGA and 20 IUGR full-term fetuses and neonates on postnatal days 1 and 4. hs-CRP, PAI-1, and S100B levels did not differ at all time points between AGA and IUGR groups. We conclude that the lack of difference in hs-CRP, PAI-1 and S100B levels, between IUGR and AGA fetuses/neonates—despite the lower birth weight, reflecting reduced fat mass in the former—might indicate more intense adipose tissue and nervous system inflammation in IUGRs. However, implication of other inflammation-related mechanisms, common in the IUGR state (e.g. preeclampsia), cannot be excluded.

## 1. Introduction

Intrauterine growth restriction (IUGR), a major cause of infant mortality and morbidity [1, 2], is strongly related to the later development of the metabolic syndrome in adult life [3–5]. The metabolic syndrome represents a cluster of cardiovascular risk factors, such as visceral obesity, lipid and glucose metabolism abnormalities, leading to type 2 diabetes mellitus and arterial hypertension [6]. Insulin resistance has been proposed to be the underlying pathogenic link between metabolic syndrome and cardiovascular disease [7–9] and is associated with a state of low-grade aseptic inflammation that precedes the onset of metabolic syndrome [10, 11]. Data suggest that, compared with appropriate for gestational age (AGA) newborns, those with low birth weight may have relatively increased visceral fat stores [12, 13], as well as elevated levels of adipocytokines, such as leptin [14–16] and visfatin [17], which link the adipose depot to insulin resistance and sensitivity, respectively, as well as to aseptic inflammation [18]. 

C-reactive protein (CRP) is considered the major acute-phase reactant in humans. It is an important first-line host defence molecule, which recognises pathogens and damaged cells and promotes their elimination by activating the complement system and mediating their phagocytic clearance [19, 20]. Moreover, using high-sensitivity assays for CRP (hs-CRP), several studies have shown elevated CRP levels in obesity, since adiposity resembles a low grade systemic inflammatory state and hs-CRP is released by adipose tissue [21, 22]. 

Plasminogen activator inhibitor-1 (PAI-1), secreted by vascular cells, is the primary physiological inhibitor of plasminogen activation in blood, contributing to thrombus formation [23]. It either promotes or prevents vascular remodelling processes, such as neointima formation, atherosclerosis, and pulmonary hypertension [24]. In addition, PAI-1 is produced by adipose tissue [25–27] and macrophages infiltrating it [28] and contributes to obesity-related insulin resistance [25].

 S100B is an acidic calcium-binding protein mainly concentrated in the central nervous system [29]. It has been reported to regulate several cellular functions (cell growth, cell structure, and energy metabolism) at physiologic concentrations, whereas it has been shown to be neurotoxic in high concentrations [30]. Elevated S100B levels have been shown to represent a marker of brain damage and hypoxic-ischemic encephalopathy in asphyxiated infants [31–34], thus possibly reflecting brain and central nervous system inflammation. 

Based on the above, we hypothesized that circulating levels of hs-CRP, PAI-1 and S100B—all markers implicated in perinatal stress physiology—probably reflecting brain and adipose tissue inflammation, might differ in IUGR compared with AGA pregnancies. Therefore, we aimed to determine their levels at time-points characteristic for intra- and extrauterine life.

## 2. Materials and Methods

### 2.1. Subjects

The study was approved by the Ethics Committee of our teaching hospital and informed consent was obtained from participating mothers. Forty parturients giving consecutively birth either to 20 AGA or 20 asymmetric IUGR full-term singleton infants were recruited. Gestational age was estimated using the date of the last menstrual period and early antenatal ultrasound. Birth weight was measured with an electronic scale. Newborns below the third customized centile for birth weight were defined as IUGR. For the calculation of the customized centiles, the principles of the Gestation-Related Optimal Weight (GROW) program [35, 36] were used. Significant determinants of birth weight (maternal height and booking weight, ethnicity, parity, gestational age and gender) were entered to adjust the normal birth weight centile limits [35].

In the IUGR group, five cases resulted from preeclampsia and seven from pregnancy-induced hypertension. In the remaining eight cases, the cause of IUGR was unclear; however, parturients suffered from chronic diseases (anaemia, type 1 diabetes mellitus, cardiac arrhythmias, and thyroiditis). Furthermore, five of the above mothers were smoking >10 cigarettes/day during the whole duration of pregnancy.

 Doppler studies were performed in the IUGR group every 10–15 days, starting from the 32nd gestational week. During each Doppler velocimetry evaluation, three consecutive measurements of the pulsatility index (PI) of the studied vessel (uterine, umbilical, cerebral arteries) were done and the mean value was recorded. Concerning uterine and umbilical arteries [37, 38], mean PI values were progressively found to be in the upper physiological limits for the corresponding gestational age in 10 cases (ranging between the 90th and the 95th percentile), while in the remaining 10 cases PI values showed increased impedance to flow, being above the 95th percentile for gestational age. Regarding middle cerebral arteries [39], Doppler studies showed resistance to be in the lower physiological limits for gestational age, indicating the initiation of blood flow redistribution process, in order to spare vital organs (brain, heart, and adrenals). Nevertheless, amniotic fluid was diminished in all IUGR cases. For the evaluation of the amniotic fluid, the largest fluid column on the vertical plane was assessed and was defined as diminished if <2 cm. Placental weights were reduced, ranging from 240 to 450 g. 

In the AGA group, mothers were healthy and were either non-smokers or abstained from smoking during pregnancy. Placentas were normal in appearance and weight. Tests for congenital infections were negative in all women of both groups and their offspring had no symptoms of intrauterine infection or signs of genetic syndromes. One- and five-minute Apgar scores were ≥8 in all IUGR cases and AGA controls. The demographic data of participating newborns and their mothers are listed in Table 1.

### 2.2. Protocol

Blood was collected from: (i) the mothers (MS) during the first stage of labor, or before receiving anesthesia in cases of elective caesarean section, (ii) the doubly clamped umbilical cords (UC-mixed arteriovenous blood), reflecting fetal state, and (iii) the neonates on postpartum day 1(N1) and 4 (N4), characterizing transition and stabilization to extrauterine life, respectively. Plasma was separated by centrifugation and was kept frozen at −80°C until assay.

### 2.3. Assays

Serum hs-CRP level determination was performed by fully mechanized latex-particle-enhanced immunonephelometric assays on the BN ProSpec nephelometer (Dade Behring, Siemens Healthcare Diagnostics, Liederbach, Germany). The intra- and interassay coefficients of variation (CVs) were less than 6% and 7%, respectively. 

PAI-1 levels were measured in citrate plasma with an enzyme-linked immunosorbent assay (Asserachrom^®^ PAI-1, Stago, Asnieres-sur-Seine, France). The minimum detectable concentration was 1.0 ng/mL. The intra- and interassay coefficients of variation were <6.0% and 7.0%, respectively. 

Serum S100B protein was analyzed with the use of the immunoluminometric assay Sangtec 100, (Sangtec Medical AB, Bromma, Sweden) and was measured by chemiluminescence on the LIAISON^®^ random access analyzer (DiaSorin, Saluggia, Italy). Sangtec 100 measures the *β*-subunit of S100 as defined by 3 monoclonal antibodies. The minimum detectable concentration of the assay was 0.02 *μ*g/L. The analysis represents the total amount of S100 and S100B in the sample, as the assay is specific for the beta-chain. The intra-and interassay coefficients of variation for S100B were less than 3.8 and 5.7%, respectively.

Finally we used data concerning serum levels of the anti-inflammatory agent cortisol which were previously determined [40].

## 3. Statistical Analysis

Data distribution was tested with the Kolmogorov Smirnov test. PAI-1, hs-CRP, and S100B levels were not normally distributed, thus nonparametric procedures (Mann-Whitney test, Friedman test, Wilcoxon Sign Rank Test, Spearman Rank correlation coefficient) were applied in the analysis. *P* values <.05 were considered significant.

## 4. Results

**Figure 1:**
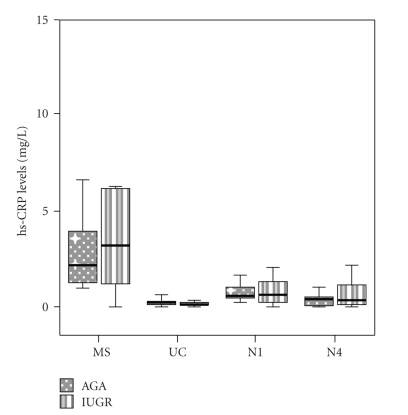
Box and whiskers plot presentation of maternal (MS), fetal (UC), neonatal day 1 (N1), and neonatal day 4 (N4) C-reactive protein (hs-CRP) levels. Each box represents the median concentration with the interquartile range. The upper and lower whiskers represent the range.

**Figure 2:**
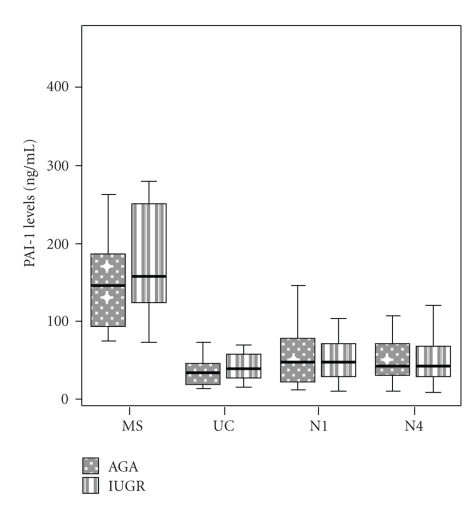
Box and whiskers plot presentation of maternal (MS), fetal (UC), neonatal day 1 (N1), and neonatal day 4 (N4) PAI-1 levels. Each box represents the median concentration with the interquartile range. The upper and lower whiskers represent the range.

**Figure 3:**
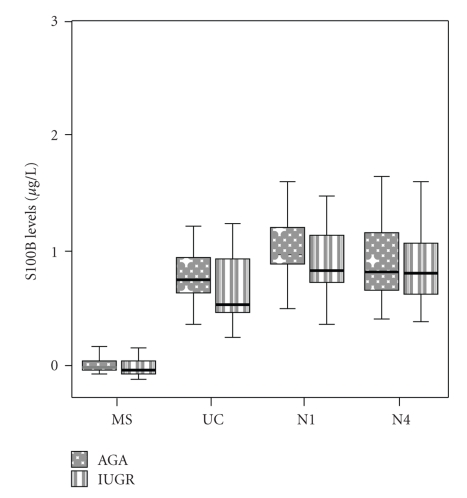
Box and whiskers plot presentation of maternal (MS), fetal (UC), neonatal day 1 (N1) and neonatal day 4 (N4) S100B levels. Each box represents the median concentration with the interquartile range. The upper and lower whiskers represent the range.

Maternal (MS), fetal (UC), day 1 (N1), and day 4 (N4) neonatal levels of inflammatory markers in IUGR cases and AGA controls are presented in Figures 1, 2, and 3. 

hs-CRP levels did not differ significantly at all time points between AGA and IUGR groups. In both groups, MS hs-CRP was significantly higher than UC, N1 and N4 values (*P* < .001, *P* = .012, and *P* = .001, resp., in the AGA group and *P* < .001, *P* = .001 and *P* = .001, resp., in the IUGR group). UC hs-CRP was significantly decreased when compared to N1 hs-CRP (*P *<.001) in the AGA group, and to N1 and N4 hs-CRP (*P * = .002 and *P * = .006, resp.,) in the IUGR group. 

PAI-1 levels did not differ significantly at all time points between AGA and IUGR groups. In both groups, MS PAI-1 was significantly higher than UC, N1, or N4 PAI-1 (*P* < .001, *P* < .001, and *P* = .003, resp., in the AGA and *P* = .004, *P* = .001, and *P* < .001, resp., in the IUGR group). No significant differences were observed among UC, N1 and N4 PAI-1 measurements, either in the AGA or the IUGR group. 

 S100B levels did not differ significantly at all time points between AGA and IUGR groups. In both groups, MS S100B was significantly lower than UC, N1 and N4 levels (*P* < .001, *P* < .001 and *P* = .001, resp., in the AGA group and *P* < .001 in all cases in the IUGR group). In both groups, N1 S100B was significantly increased when compared to respective UC S100B (*P* = .004 for both groups). 

Statistically significant correlations among inflammatory markers and cortisol at all time points in AGA and IUGR neonates and their mothers are reported in Table 2. Thus, positive correlations were documented between fetal PAI-1 levels and maternal age (in the IUGR group), as well as between maternal and fetal cortisol levels (in both groups). On the other hand, negative correlations were found (in the IUGR group) between maternal cortisol and fetal PAI-1 levels, as well as fetal cortisol and maternal/fetal S100B and fetal PAI-1 levels.

## 5. Discussion

The results of this study indicate that inflammatory markers, implicated in perinatal stress physiology, such as hs-CRP, PAI-1 and S100B, determined in the plasma of AGA and IUGR mother/infant pairs at birth and on postnatal days 1 and 4 did not differ between the two studied groups.

Maternal hs-CRP levels were found significantly higher than fetal and neonatal ones in both studied groups. It has been previously shown, that maternal CRP values consistently increase throughout pregnancy as compared to the nonpregnant state, culminating during labour [41, 42]. Furthermore, it is known that CRP does not cross the placental barrier [43], justifying the significantly lower levels found in fetuses and neonates. 

On the other hand, CRP is a marker of inflammation produced by adipose tissue [21]. Considering that IUGR babies have by definition lower amounts of total fat mass as compared to AGA babies, the lack of difference in CRP levels between these two groups, could suggest a more intense inflammatory state in the adipose tissue of the former. The gradual increase of CRP in both studied groups from UC to N1, and N4 could possibly be attributed to adaptational stress [44–46] to extrauterine life. CRP is produced by pulmonary alveolar macrophages (PAM), among other immune response cells [47], and studies in animals have shown the rapid increase of PAM during postnatal development [48]. Thus, it is possible that the major source of postpartum CRP production is the constantly increasing PAM population in the neonatal lung.

 Furthermore, we found that in both studied groups, maternal PAI-1 levels were significantly higher than fetal and neonatal ones. Taken that adipose tissue is an important source of PAI-1 production [49], this could be attributed to its higher amounts in adults as compared to neonates. 

The lack of difference in PAI-1 levels between IUGR and AGA groups could speculatively be attributed, as for CRP, to a state of more intense adipose tissue-related inflammation in the IUGR, as compared to the AGA neonates. In this respect, we have previously documented that circulating levels of the adipocytokines leptin and visfatin are relatively increased in IUGR neonates, despite their reduced total fat mass [17, 40]. Moreover, CRP has been shown to induce PAI-1 expression [50–53]. In addition, other studies have documented that PAI-1 is mainly produced by visceral fat [49], which is proportionally higher in the IUGR than the AGA neonates [12, 13]. 

However, another explanation could be considered. Thus, dysfunctional vascular endothelium, characterizing preeclampsia resulting to IUGR, is associated with increased PAI-1 release [54]. In this respect, the lack of significant differences in PAI-1 levels between IUGR and AGA groups in the present study could possibly rely to the fact that only five out of 20 IUGR pregnancies were complicated by preeclampsia.

Furthermore, smoking in pregnancy, apart from being a risk factor for IUGR [55], has a deleterious effect on vascular endothelium, resulting in endothelial dysfunction, which again is associated with PAI-1 production [56]. The fact that PAI-1 levels were similar in both groups of this study could be attributed to the limited number of IUGR mothers smoking during pregnancy.

In both groups, maternal S100B levels were significantly lower than fetal and neonatal ones (N1 and N4), while N1 S100B levels were significantly higher than respective UC ones. S100B has a neurotrophic effect during both development and nerve regeneration [57–59]. Respectively, Gazzolo et al. documented higher S100B concentrations in children during the first year of life and in adolescence, as compared to adults, thus documenting its neurotrophic role [57]. This fact might explain on one hand, the low S100B levels in mothers, and on the other, the gradual increase of S100B levels from N1 to N4.

The lack of significant differences in the levels of S100B between the two studied groups in the three perinatal time points could possibly be attributed to the brain-sparing phenomenon in cases of IUGR that ensures nutrient supply to vital organs [60, 61]. Thus, glial cells, mainly producing S100B, remain unaffected by malnutrition [57]. Alternatively, Florio et al. documented increased levels of S100B in urine samples from IUGR neonates, with highest levels in those with abnormal neurological outcome [62]. Indeed, S100B is also a marker of hypoxia-induced brain damage [29, 62]. Nevertheless, the IUGR group in this study consisted of asymmetrical IUGR babies, with good Apgar scores at birth and no neurologic sequelae in the short term, whatsoever. 

Furthermore, this study showed that in the IUGR group, UC PAI-1 positively correlated with maternal age. Previous studies [63, 64] have reported significantly higher placental PAI-1 levels in IUGR pregnancies with or without preeclampsia. Taken that older gravidas are at increased risk of IUGR pregnancies complicated by preeclampsia and abnormal placentation [65], the above finding could be justified. 

On the other hand, controversial reports in the literature also associate very young maternal age groups with preeclampsia [66–72]. Nevertheless, the present study did not comprise in the IUGR group such individuals, since the age range of the included mothers was 24–38 years. 

The positive correlation of maternal and fetal cortisol levels found in both groups of our study is probably due to placental transfer [73, 74].

As cortisol has a well-known anti-inflammatory effect, the negative correlations of MS cortisol with UC PAI-1, as well as UC cortisol with MS S100B, UC S100B, and UC PAI-1 in the IUGR group (Table 2), further enhance the hypothesis for the inflammation-mediating role of S100B and PAI-1. Besides, studies have also documented the reduction of S100B in the presence of corticosteroids [75, 76]. 

In conclusion, the absence of difference in hs-CRP and PAI-1 levels, between IUGR cases and AGA controls, possibly indicates an accentuated secretion of these markers from the adipose tissue in the former, who despite their lower birth weight, bear a relatively increased amount of visceral fat as compared to the latter. On the other hand, the lack of difference in S100B levels between these two groups possibly reflects the brain-sparing effect occurring in IUGR cases. Moreover, cortisol remains a major anti-inflammatory compound that counteracts the effect of the above mentioned inflammatory markers. Thus, our findings could probably support the concept of aseptic inflammation in IUGR fetuses and neonates. However, implication of other inflammation-related mechanisms, common in the IUGR state (e.g. preeclampsia) cannot be excluded.

## Figures and Tables

**Table 1:** Demographic data of participating infants (appropriate for gestational age-AGA and intrauterine growth restricted-IUGR) and of their mothers.

	AGA MEAN±SD	IUGR MEAN±SD	*P* value
Maternal age (years)	31 ± 4	31 ± 7	NS
BMI (pre-pregnancy)	22.0 ± 6.1	22.3 ± 6.3	NS
Gestational age (weeks)	38.9 ± 1	37.9 ± 1.38	.016
Birthweight (g)	3167 ± 258	2377 ± 262	<.001

*Gender N (%)*			NS
Male	8 (40)	7 (35)	
Female	12 (60)	13 (65)	

*Mode of delivery N (%)*			NS
Vaginal delivery	9 (45)	9 (45)	
Elective caesarean section	11 (55)	11 (55)	

**Table 2:** Significant correlations among hs-CRP, PAI-1, S100B and cortisol levels in IUGR and AGA groups of the MS, UC, N1, and N4 samples.

	IUGR	AGA
UC PAI-1 versus maternal age	*r* = 0.473,	NS
	*P* = .041	
MS CORTISOL versus UC CORTISOL	*r* = 0.824,	*r* = 0.748,
	*P* < .001	*P* < .001
MS CORTISOL versus UC PAI-1	*r* = −0.581,	NS
	*P* = .011	
UC CORTISOL versus MS S100B	*r* = −0.494,	NS
	*P* = .037	
UC CORTISOL versus UC S100B	*r* = −0.598,	NS
	*P* = .009	
UC CORTISOL versus UC PAI-1	*r* = −0.507,	NS
	*P* = .038	
